# Evaluating the Effects of Omega-3 Polyunsaturated Fatty Acids on Inflammatory Bowel Disease via Circulating Metabolites: A Mediation Mendelian Randomization Study

**DOI:** 10.3390/metabo13101041

**Published:** 2023-09-28

**Authors:** Xiaojing Jia, Chunyan Hu, Xueyan Wu, Hongyan Qi, Lin Lin, Min Xu, Yu Xu, Tiange Wang, Zhiyun Zhao, Yuhong Chen, Mian Li, Ruizhi Zheng, Hong Lin, Shuangyuan Wang, Weiqing Wang, Yufang Bi, Jie Zheng, Jieli Lu

**Affiliations:** 1 Department of Endocrine and Metabolic Diseases, Shanghai Institute of Endocrine and Metabolic Diseases, Ruijin Hospital, Shanghai Jiao Tong University School of Medicine, Shanghai 200025, China; 2 Shanghai National Clinical Research Center for Metabolic Diseases, Key Laboratory for Endocrine and Metabolic Diseases of the National Health Commission of the PR China, Shanghai Key Laboratory for Endocrine Tumor, State Key Laboratory of Medical Genomics, Ruijin Hospital, Shanghai Jiao Tong University School of Medicine, Shanghai 200025, China; 3 MRC Integrative Epidemiology Unit (IEU), Bristol Medical School, University of Bristol, Oakfield House, Oakfield Grove, Bristol BS8 2BN, UK

**Keywords:** eicosapentaenoic acid, inflammatory bowel disease, Mendelian randomization, mediation, omega-3 polyunsaturated fatty acids

## Abstract

Epidemiological evidence regarding the effect of omega-3 polyunsaturated fatty acid (PUFA) supplementation on inflammatory bowel disease (IBD) is conflicting. Additionally, little evidence exists regarding the effects of specific omega-3 components on IBD risk. We applied two-sample Mendelian randomization (MR) to disentangle the effects of omega-3 PUFAs (including total omega-3, α-linolenic acid, eicosapentaenoic acid (EPA), or docosahexaenoic acid (DHA)) on the risk of IBD, Crohn’s disease (CD) and ulcerative colitis (UC). Our findings indicated that genetically predicted increased EPA concentrations were associated with decreased risk of IBD (odds ratio 0.78 (95% CI 0.63–0.98)). This effect was found to be mediated through lower levels of linoleic acid and histidine metabolites. However, we found limited evidence to support the effects of total omega-3, α-linolenic acid, and DHA on the risks of IBD. In the *fatty acid desaturase 2* (*FADS2*) region, robust colocalization evidence was observed, suggesting the primary role of the *FADS2* gene in mediating the effects of omega-3 PUFAs on IBD. Therefore, the present MR study highlights EPA as the predominant active component of omega-3 fatty acids in relation to decreased risk of IBD, potentially via its interaction with linoleic acid and histidine metabolites. Additionally, the *FADS2* gene likely mediates the effects of omega-3 PUFAs on IBD risk.

## 1. Introduction

Inflammatory bowel disease (IBD) is a group of chronic inflammatory disorders affecting the gastrointestinal tract, and its prevalence has increased worldwide, reaching up to 0.5% of the general population in the western world [1,2]. The two primary types of IBD are Crohn’s disease (CD) and ulcerative colitis (UC), each with different clinical and histopathological characteristics [3]. The economic burden of IBD is substantial, with over €4.6 billion in annual medical costs in Europe and US$6 billion in the USA, putting a strain on healthcare systems and resources [2]. To alleviate this burden, a comprehensive approach is needed, including the development of preventive care to delay the progression of this disease. Omega-3 polyunsaturated fatty acids (PUFAs) are commonly used nutritional supplements and show beneficial effects on coronary heart disease [4] and asthma [5]. Due to their anti-inflammatory properties, PUFAs have been proposed as potential targets for preventing and treating autoimmune diseases [6]. Omega-3 PUFAs can be quantified based on a shift in the signal induced by the position of the omega-3 double bond. The sum of concentrations of α-linolenic acid, eicosapentaenoic acid (EPA), docosahexaenoic acid (DHA), and other omega-3 PUFAs is expressed as total omega-3 fatty acids. Long-chain omega-3 PUFAs (EPA and DHA) are derived from α-linolenic acid through a series of elongation, desaturation, and β-oxidation events during fatty acid metabolism. The *fatty acid desaturase 2* (*FADS2*) gene encodes delta-6 desaturase and plays a key regulatory role in this metabolism process [7].

In randomized controlled trials (RCTs), EPA and DHA have often been combined as the active components of omega-3 fatty acids and consumed together, despite their distinct molecular functions and clinical impacts [8]. Daily supplementation with EPA and DHA were reported to be effective in reducing the clinical relapse of CD [9]. However, in the large-scaled vitamin D and omega 3 trial (VITAL) with approximately five years of randomized follow-up, fish oil containing EPA and DHA did not significantly reduce the rate of a composite outcome consisting of rheumatoid arthritis, IBD, autoimmune thyroid disease, and all other autoimmune diseases [10]. Moreover, there was a lack of detailed information on IBD in this study. Additionally, observational studies did not provide convincing and consistent evidence of the relationship between dietary intakes of omega-3 PUFAs and the risk of IBD [11,12,13]. Information on usual diet relied on self-reported dietary questionnaires, which may produce errors or bias in recall. The existing evidence makes it challenging to confirm the causal effect of omega-3 PUFAs on IBD; and identify the key supplement among the omega-3 PUFA component (α-linolenic acid, EPA, and DHA) that may exhibit the protective effect.

Mendelian randomization (MR) is an approach that could estimate causal effect of an exposure on an outcome and overcome issues related to residual confounding or reverse causality [14]. Moreover, this method allows for investigating the effects of each omega-3 PUFA component on IBD, which may be challenging to achieve in an RCT setting. Recently, He et al. reported that total omega-3 fatty acid had a protective effect against increased UC risk instead of CD [15], but the evidence on IBD was not addressed. In addition, their analysis involved only 21 omega-3 instruments after eliminating SNPs associated with potential confounders and outcomes, which might have reduced the power of the analysis. More critically, some instruments in key regulatory genes such as *FADS2* gene were eliminated, which may have important influences on the reliability of the findings. Meanwhile, there remains a knowledge gap in evaluating the separate biological effects of α-linolenic acid, EPA, and DHA, with their metabolic mechanisms being unexplored.

In this study, we aimed to explore the effects of omega-3 PUFAs (i.e., total omega-3, α-linolenic acid, EPA, and DHA) on the risk of IBD and its subtypes, and the potential metabolic pathways linking omega-3 PUFAs with IBD. Given the central role of the *FADS2* gene in omega-3 PUFAs’ metabolism, further analyses in this specific region were essential through genetic colocalization. This approach allowed us to assess whether there were shared causal variants within the *FADS2* gene region that could influence both omega-3 PUFAs and IBD risk [16].

## 2. Materials and Methods

### 2.1. Study Design 

**Figure 1:**
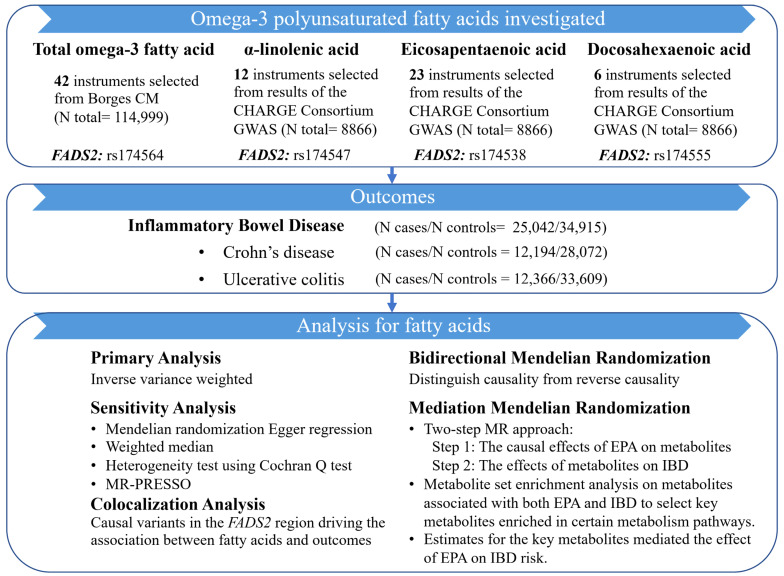
Study design of this MR study.

A schematic overview of the study design was detailed in Figure 1. We employed the univariable MR analysis to assess whether total omega-3 fatty acid, α-linolenic acid, EPA, and DHA showed causal effects on IBD and its subtypes (CD and UC), using summary-level data from publicly available genome-wide association studies (GWASs). Colocalization analysis was further conducted in the *FADS2* gene region to test for pleiotropic effect and investigate the underlying mechanisms. A bidirectional MR analysis was applied to estimate the effect of genetic liability to IBD on omega-3 PUFAs. Mediation MR analysis estimated the effect of potential metabolites linking omega-3 PUFAs with the IBD. All datasets were publicly available, and ethical approval was acquired for all original studies.

### 2.2. Data Sources and Genetic Instruments for Omega-3 PUFAs

Single-nucleotide polymorphisms (SNPs) associated with total omega-3 fatty acid were derived from UK Biobank, which collected deep genetic and phenotypic data from approximately 500,000 individuals aged between 40 and 69 [17]. Genetic associations of α-linolenic acid, EPA, and DHA were obtained from a GWAS meta-analysis in 8866 participants of European ancestry from the Cohorts for Heart and Aging Research in Genomic Epidemiology (CHARGE) Consortium [18]. Details of the data sources and sample sizes of the exposures are listed in the Table S1.

In this study, the genetic variants that showed robust association with total omega-3 fatty acid (with genetic association *p* value < 5 × 10^−8^) and showed independence (with linkage disequilibrium (LD) r^2^ < 0.01 in European ancestry) were selected as candidate instruments. Given the limited sample size of the α-linolenic acid, EPA, and DHA GWASs, a slightly more relaxing threshold (*p* < 5 × 10^−6^) was used to select instruments for these exposures. After harmonization with outcome data and removing palindromic or mismatching alleles, 42 independent SNPs for total omega-3 fatty acid, 12 independent SNPs for α-linolenic acid, 23 independent SNPs for EPA, and 6 independent SNPs for DHA were selected as instruments (Figure S1). One SNP was selected to represent the effect of each omega-3 PUFA in the *FADS2* region (rs174564 for total omega-3 fatty acid, rs174547 for α-linolenic acid, rs174538 for EPA, and rs174555 for DHA; all these SNPs are in strong LD to each other (LD r^2^ > 0.7), which represents the same signal in this region).

### 2.3. Outcome Data Sources 

Summary statistics for IBD were obtained from the study by the International Inflammatory Bowel Disease Genetics Consortium (IIBDGC) [19], which contained a total of 59,957 European participants (cases/controls for IBD: 25,042/34,915; UC: 12,366/33,609; CD: 12,194/28,072). All the cases were diagnosed using accepted endoscopic, histopathological and radiological criteria and all the control samples were obtained from the Understanding Society Project.

### 2.4. Metabolite Data Sources

The full GWAS summary statistics of the 974 circulating metabolites were derived from the IEU OpenGWAS database with GWAS identifier met-a, met-b, met-c, and met-d.

### 2.5. Statistical Analysis

#### 2.5.1. Two-Sample MR Analysis

The inverse weighted variance (IVW) method was used as a primary analysis to estimate the causal effects of omega-3 PUFAs on IBD and its subtypes, which were calculated by a weighted linear regression of the instrument–outcome association estimates on the instrument–exposure association estimates assuming that all genetic variants were valid instruments [14].

MR analysis had several assumptions. The genetic instruments need to (1) be robustly associated with the exposure (“relevance”), (2) be independent of potential confounders of the instrument–outcome association (“exchangeability”), and (3) only affect the outcome through the exposure being tested and not through alternative pathways (that is, through pleiotropy; “exclusion restriction”).

The relevance MR assumption was assessed from the mean F statistics within univariable MR, which was greater than ten for every instrument–exposure association, demonstrating the small possibility of weak instrumental variable bias [20].

We attempted using colocalization to test exchangeability MR assumption whether SNPs associated with two traits are possibly in LD, or a single shared signal (colocalization) [16]. The *FADS2* gene encoded key fatty acid desaturase enzymes, which are pivotal for omega-3 PUFA biosynthesis. Therefore, we performed colocalization analyses in this gene region. A generated colocalization posterior probability greater than 0.70 indicated the same variant causal for both traits and indirectly denied the possibility of “exchangeability”. 

To check for violation of the “exclusion restriction” assumption of MR and assess pleiotropy, we made different assumptions regarding MR instrument validity using several sensitivity analyses, that included the weighted median method which permitted up to 50% of the information in the MR analysis to come from invalid instruments [21], and the MR-Egger approach which accounted for pleiotropy [22]. The MR pleiotropy residual sum and outlier (MR-PRESSO) method [23], together with MR-Egger intercept could be used to examine the level of horizontal pleiotropy, and reduce the level of horizontal pleiotropy via outlier removal. In addition, heterogeneity of the estimates was detected using Cochran’s Q [24]. A leave-one-out analysis was conducted by removing each SNP from the analysis in turn and performing an IVW method on the remaining SNPs to assess the potential influence of a particular variant on the estimates [25].

We also sought to evaluate whether there was a reverse causal effect where liability to IBD consequently altered the levels of omega-3 PUFAs by performing a bidirectional MR analysis [26]. Therefore, we took independent instruments robustly associated with IBD, CD, and UC (*p* < 5 × 10^−8^) as exposures to assess their effect on total omega-3 fatty acid, α-linolenic acid, EPA, and DHA, respectively.

#### 2.5.2. Mediation MR Analysis Linking EPA with IBD via Metabolites

We further estimated the mediation effects of circulating metabolites linking EPA with IBD risk. We used a novel analytical pipeline that integrated mediation MR with metabolite set enrichment analyses. First, we used a two-step MR approach to: (1) assess the causal effect of EPA on 974 potential metabolites (step 1) that have publicly available GWAS datasets in the IEU OpenGWAS database, which selected 237 metabolites with FDR < 0.05; and (2) estimate the effect of 237 metabolites on IBD (step 2), which further selected 211 metabolites associated with both EPA and IBD as candidate mediation metabolites. Second, we performed the metabolite set enrichment analysis on the 211 selected candidate metabolites, which aimed to select key metabolites enriched in certain metabolic pathways (Figure S1). For the metabolites that showed evidence of enrichment in the enrichment analysis, we further performed multivariable MR (MVMR) to determine their mediation effects on IBD which was adjusted for the effect of EPA [27]. We used IVW as our main approach to estimate the effect of EPA on the metabolites (β1). Additionally, MVMR was applied to estimate: (1) the effect of each metabolite on risk of IBD with adjustment for the genetic effect of EPA (β2); and (2) the direct effect of EPA on IBD with adjustment for each mediator individually (βdirect). To calculate the indirect mediation effect of EPA on IBD outcome, we used the difference of coefficients method as our main method, i.e., the casual effect of EPA on outcomes via metabolites (βtotal − βdirect). The total effect was the estimate of EPA on IBD in univariable MR (βtotal). Thus, the proportion of the total effect mediated by each metabolite was separately estimated by dividing the indirect effect by the total effect ((βtotal − βdirect)/βtotal). Standard errors were derived by using the delta method, using effect estimates obtained from 2SMR analysis.

Univariable, bidirectional, and multivariable MR analyses were considered significant with a 2-sided *p* ≤ 0.05. Metabolites associated with omega-3 PUFAs or IBD were considered significant with an FDR < 0.05. Enrichment analysis was performed using the online MetaboAnalyst software (version 5.0, Mcgill University, Montreal, QC, Canada; https://www.metaboanalyst.ca, accessed on 17 November 2022) [28]. All analyses were performed using ‘TwoSampleMR’ and ‘MR-PRESSO’ package in R Software 3.6.0.

## 3. Results

We selected 42, 12, 23, and 6 SNPs as instruments to proxy life-long effect of total omega-3 fatty acid, α-linolenic acid, EPA, and DHA, respectively. In bidirectional MR, there were 117, 89, and 62 independent instruments incorporated for IBD, CD, and UC, respectively. Mean F statistics of the exposures ranged from 29.82 to 262.21 indicating that the MR estimates were not likely to be influenced by weak instrument bias (Table S2).

### 3.1. Genetically Predicted Omega-3 PUFAs on Risk of IBD (Including CD and UC)

Table 1 shows the effects of omega-3 PUFAs on IBD risks. Considering total omega-3 fatty acid as a whole, little evidence indicated its protective effect on IBD risk (odds ratio (OR) of IVW, 0.94; 95% confidence interval (CI), 0.82–1.07). Meanwhile, higher concentrations of α-linolenic acid showed a potential effect on increasing risk of IBD, although the evidence was weaker due to the wide confidence interval (OR of IVW, 1.54; 95% CI, 0.72–3.29). In contrast, genetically increased levels of EPA showed a causal effect on the lower risk of IBD (OR of IVW, 0.78; 95% CI, 0.63–0.98). There was little evidence for the presence of heterogeneity (Cochran’s Q-test *P_h_* = 0.10), pleiotropy (MR-Egger intercept *P_intercept_* = 0.97), or any outliers (MR-PRESSO *P* of global test = 0.099). Estimated effect was consistent using the weighted median approach (OR, 0.59; 95% CI, 0.45–0.78). However, there was little evidence to support the effect of DHA on IBD (OR of IVW, 1.05; 95% CI, 0.86–1.28).

**Figure 2:**
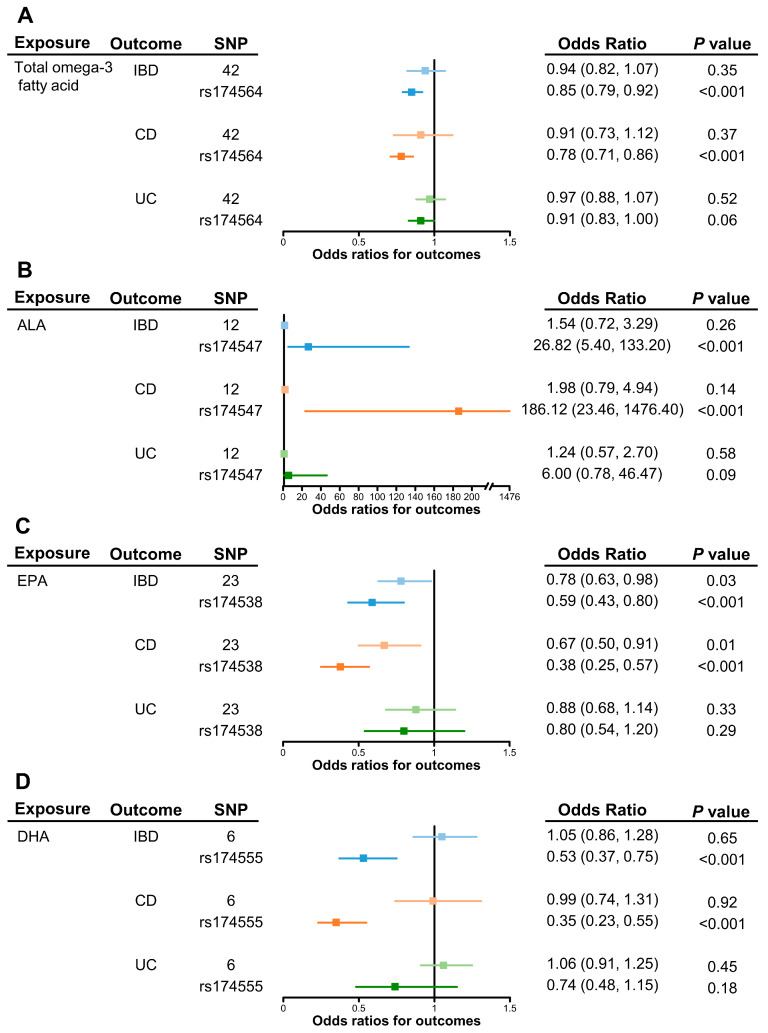
Causal effects of omega-3 polyunsaturated fatty acids on inflammatory bowel disease as a whole, on Crohn’s disease, and ulcerative colitis or via the *FADS2* gene cluster. Univariable causal effects of (**A**) total omega-3, (**B**) α-linolenic acid, (**C**) EPA, and (**D**) DHA on investigated outcomes (light shades of blue, orange and green). Causal effects of each fatty acid on investigated outcomes via the *FADS2* gene (blue, orange and green). Abbreviations: ALA, α-linolenic acid; CD, Crohn’s disease; DHA, docosahexaenoic acid; EPA, eicosapentaenoic acid; *FADS2*, *fatty acid desaturase 2*; IBD, inflammatory bowel disease; UC, ulcerative colitis.

The results of the primary MR analyses of CD and UC are presented in Figure 2. Results of sensitivity analyses are listed in Table S3. In consistent with the IBD results, there was little evidence to support the effects of total omega-3 fatty acid, α-linolenic acid, and DHA on the risk of CD and UC (Figure 2A,B,D). Meanwhile, increased levels of genetically proxied EPA still showed a strong effect on a lower risk of CD (OR of IVW, 0.67; 95% CI, 0.50–0.91), but with little effect on UC (OR of IVW, 0.88; 95% CI, 0.68–1.14) (Figure 2C).

### 3.2. Sensitivity Analysis in FADS2 Gene Region

As shown in the leave-one-out analyses, the MR estimates of omega-3 PUFAs on IBD, CD, and UC were mainly driven by SNP effects in the *FADS2* gene region (rs174564 for total omega-3 fatty acid, rs174547 for α-linolenic acid, rs174538 for EPA, and rs174555 for DHA) (Figure S2). As shown in Figure 2, the MR results of the single *FADS2* SNP showed the causal effects of total omega-3 fatty acid, EPA, and DHA on lower risk of IBD. The ORs (95% CI) were 0.85 (0.79–0.92), 0.59 (0.43–0.80), and 0.53 (0.37–0.75), respectively. On the contrary, α-linolenic acid showed a strong effect on the increasing risk of IBD (OR, 26.82; 95% CI, 5.40–133.20). 

As for IBD subtypes, the *FADS2* gene showed a stronger effect on lowering the risk of CD (ORs (95% CI) were 0.78 (0.71–0.86) for total omega-3, 0.38 (0.25–0.57) for EPA, and 0.35 (0.23–0.55) for DHA) but was absent for UC. Meanwhile, a *FADS2* single-SNP in α-linolenic acid had a positive effect on increasing CD risk (OR, 186.12; 95% CI, 23.46–1476.40), but with less effect on UC risk.

**Figure 3:**
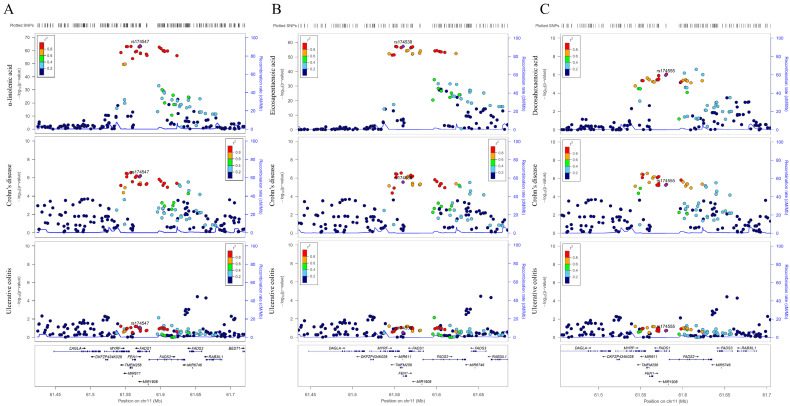
Regional association plots of α-linolenic, eicosapentaenoic, and docosahexaenoic acids with Crohn’s disease and ulcerative colitis in the *FADS2* region. (**A**) Regional plots of α-linolenic acid and Crohn’s disease and ulcerative colitis in the *FADS2* region without conditional analysis. (**B**) Regional plots of eicosapentaenoic acid and Crohn’s disease and ulcerative colitis in the *FADS2* region without conditional analysis. (**C**) Regional plots of docosahexaenoic acid and Crohn’s disease and ulcerative colitis in the *FADS2* region without conditional analysis. This figure was obtained from http://locuszoom.org/. Abbreviations: *FADS2*, *fatty acid desaturase 2*.

Aligning with the MR estimates of a single-SNP in the *FADS2* region, we observed compelling evidence of colocalization for α-linolenic acid with CD (colocalization probability, 98.90%), but with little evidence for UC (colocalization probability, 2.61%; Figure 3A). A similar pattern of colocalization evidence was observed for EPA (colocalization probability of CD, 98.80%; colocalization probability of UC, 2.44%; Figure 3B), as well as DHA (colocalization probability of CD, 94.50%; colocalization probability of UC, 6.56%; Figure 3C). Collectively, colocalization analyses further supported distinct effects of omega-3 PUFAs on CD and UC.

### 3.3. Effects of Genetic Liability to IBD, CD, and UC on the Levels of Omega-3 PUFAs

We further estimated whether genetic liability to IBD was a causal factor on changing levels of omega-3 PUFAs using bidirectional MR. There was little evidence to suggest the causal effect of genetic liability to IBD and CD on omega-3 PUFAs by using the IVW method (Table 2). However, genetic liability to UC showed an effect on lowering levels of DHA (β −0.05 (95% CI −0.09, −0.002)).

### 3.4. Mediation MR of EPA, Metabolites, and IBD Risk

**Figure 4:**
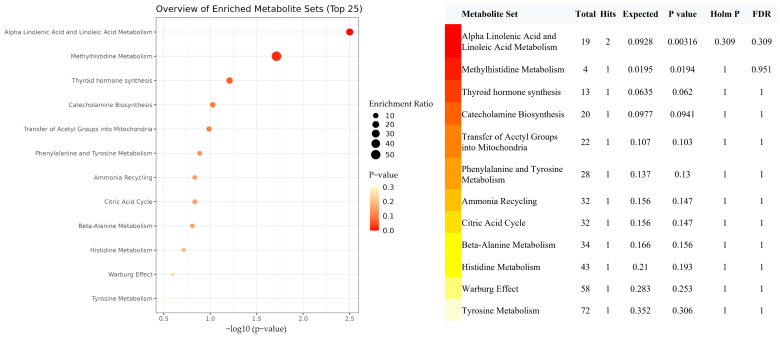
Metabolite set enrichment analysis of 211 selected candidate metabolites associated with both EPA and risk of IBD. The figure shows a graphical representation of the pathway-associated metabolite sets by enrichment analysis in the effect of EPA on IBD. Abbreviations: EPA, eicosapentaenoic acid; IBD, inflammatory bowel disease.

Given that genetically predicted increased EPA had significant benefit on lowering IBD risks, we further estimated whether there were some metabolites or metabolic pathways linking the EPA with IBD risk. For 211 candidate mediation metabolites (selected by the two-step MR described in the Section 2), metabolite set enrichment analysis indicated that α-linolenic acid and linoleic acid metabolism, and methylhistidine metabolism were the top two metabolic pathways that have been significantly enriched (Figure 4). DHA, linoleic acid, and histidine were major metabolites determined in the two pathways, respectively. 

**Figure 5:**
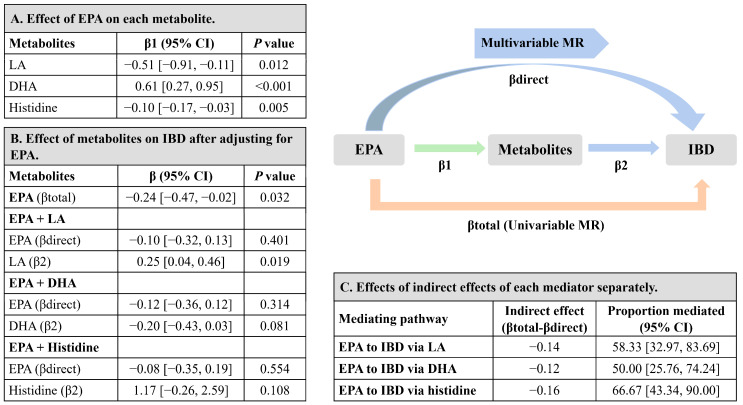
Estimates for the metabolites that mediated the effect of EPA on the risk of IBD. (**A**) MR-estimated effects of EPA on each intermediate metabolite (linoleic acid, DHA, and histidine) separately, presented as β with 95% CI. (**B**) MR-estimated effects of each intermediate metabolite separately on IBD after MVMR adjustment for EPA, presented as β with 95% CI. (**C**) MR-estimated effects of indirect effects of each intermediate metabolite separately, by using the difference of coefficients method with delta method-estimated 95% CIs. MR-estimated proportions mediated (%) are presented with 95% CIs. The sum of proportions mediated (%) were higher than 100%, due to the strong correlation among these intermediate metabolites (linoleic acid, DHA, and histidine). Abbreviations: CI, confidence interval; DHA, docosahexaenoic acid; EPA, eicosapentaenoic acid; IBD, inflammatory bowel disease; linoleic acid, linoleic acid; MR, Mendelian randomization; MVMR, multivariable Mendelian randomization.

The effect of EPA on each intermediate metabolite (linoleic acid, DHA, and histidine) is shown in Figure 5A, higher levels of EPA were associated with lower linoleic acid (β, −0.51; 95% CI −0.91, −0.11), higher DHA (β, 0.61; 95% CI 0.27, 0.95), and lower histidine (β, −0.10; 95% CI −0.17, −0.03). The effect of each intermediate metabolite on IBD risk was separately adjusted for the EPA effect in the MVMR model, presented as β with 95% CI and was shown in Figure 5B. Linoleic acid and histidine showed effects on increasing risk of IBD, although the result for histidine was with a wide confidence interval. Figure 5C displays the proportion of the mediation effect of EPA on IBD explained by each intermediate metabolite separately. Linoleic acid explained 58.33% (95% CI 32.97%, 83.69%) of the total effect of EPA on IBD, while DHA explained 50.00% (95% CI 25.76%, 74.24%). Histidine explained 66.67% (95% CI 43.34%, 90.00%) of the total effect. Given the large proportion of mediation of these intermediate metabolites, the direct effects of EPA on IBD were massively attenuated after conditioning on each of the intermediate metabolites (Figure 5B).

## 4. Discussion

The present study employed a comprehensive analysis using MR to strengthen the inferences regarding the effects of different omega-3 PUFAs (including total omega-3, α-linolenic acid, EPA, and DHA) on IBD risk. We provided evidence supporting that increased levels of EPA are causally associated with a lower risk of IBD and CD, but the effect on UC is relatively weaker. The mediation MR analysis further suggested that EPA may influence IBD via α-linolenic acid, linoleic acid and methylhistidine metabolism pathways. Linoleic acid and histidine were estimated to mediate the effect of EPA on IBD. However, we found limited evidence to support the effects of total omega-3, α-linolenic acid, and DHA on the risk of IBD. Furthermore, leave-one-out, single-locus, and colocalization analyses indicated that the effects of omega-3 PUFAs on IBD were massively driven by SNP effects in the *FADS2* gene region. Therefore, desaturation steps during omega-3 PUFAs’ biosynthesis might play a critical role in the relationship between omega-3 PUFAs and IBD. Meanwhile, higher genetic liability to UC might be associated with lower levels of DHA, potentially indicating a weaker absorption or abnormal metabolism of omega-3 PUFAs in UC. Collectively, our results suggest that supplementation with EPA (rather than α-linolenic acid or DHA) might be a more effective strategy to prevent the onset of IBD, especially CD, rather than UC with high probability of weak absorption or abnormal metabolism on omega-3 PUFAs. These findings shed light on the potential differential impacts of specific omega-3 PUFAs on IBD risk and highlight the importance of considering individual PUFA components in designing prevention strategies for this complex disease.

Previous systematic reviews and meta-analysis of RCTs have not yielded firm recommendations regarding the usefulness of omega-3 PUFAs in treating IBD [29,30]. In a study that included 19 RCTs, the results showed no significant benefits of omega-3 PUFA supplementation in maintaining remission of disease [29]. Another study of 9 RCTs, found insufficient data to support the routine use of omega-3 fatty acids for the maintenance of remission in CD and UC [30]. Similarly, a prospective investigation in the Nurses’ Health Study cohort reported that the risk of IBD was not influenced by long-term intake of omega-3 PUFAs [31]. Meanwhile, our findings showed weak evidence of protective effects of genetically predicted higher total omega-3 fatty acid against the risk of IBD and its subtypes (both CD and UC) by using MR analysis. In spite of the known anti-inflammatory properties of omega-3 PUFAs, attributed to their ability to reduce the production of cytokines [32,33] and C-reactive protein (CRP) [34], the available data provided less convincing evidence to support the use of omega-3 PUFAs in the prevention or treatment of IBD. One plausible explanation for these findings is that total omega-3 fatty acid comprises various fatty acids with different carbon chain lengths, bond saturation, and diverse biochemical mechanisms [35]. This complexity may lead to an overall effect of total omga-3 fatty acid that is diminished or challenging to decipher in relation to IBD and its subtypes. Hence, the specific roles and effects of individual omega-3 PUFAs, such as EPA and DHA, need to be explored more comprehensively to understand their potential benefits in IBD management.

α-linolenic acid serves as a substrate for other essential omega-3 PUFAs in the body. In our study, genetically predicted α-linolenic acid levels showed a trend toward an increased risk of IBD, although the statistical power of the analysis was relatively low. Observational studies have also provided inconclusive evidence regarding the relationship between α-linolenic acid and IBD. For instance, a case-control study has reported higher dietary α-linolenic acid intakes in newly diagnosed UC patients compared with healthy controls [12]. However, in consistent with our findings, previous studies did not find any association between higher dietary intake of α-linolenic acid and an increased risk of IBD [36,37]. Well powered studies are needed to investigate the effect of α-linolenic acid on IBD and other autoimmune diseases in the future.

EPA and DHA are the main components of long-chain omega-3 fatty acids, which are derived from α-linolenic acid through a series of elongation and desaturation steps and β-oxidation. The beneficial effects of EPA and DHA have been investigated as a combination or as part of omega-3 supplementation in observational studies and experimental trials. However, the distinct effects of EPA and DHA on the risk of IBD have been relatively unexplored. In our study, we conducted separate evaluations and found evidence suggesting that increased levels of EPA were associated with a lower risk of IBD and CD. 

Interestingly, our findings indicate that EPA might play a more important role than DHA in relation to IBD risk. Although direct comparative studies on the effects of EPA and DHA on IBD risk are limited, other research has provided insights that align with our results [38]. In twenty-one asthmatic adults, EPA reduced the production of interleukin-1b and tumor necrosis factor from alveolar macrophages to a much greater extent than DHA [39]. Meanwhile, the Cardiovascular Health Study reported that plasma phospholipid EPA, but not DHA, was associated with lower concentrations of CRP [40]. These findings, when integrated with our results, suggest that EPA may be more relevant for prevention of IBD.

We further demonstrated that the protective effect of EPA on risk of IBD was mainly influenced by α-linolenic acid, linoleic acid, and methylhistidine metabolism pathways. These findings are consistent with a previous study that has indicated that krill oil, rich in omega-3 PUFAs, exerts an inhibitory effect on histidine metabolism, leading to attenuated intestinal inflammation [41]. Moreover, significantly increased levels of histidine have been found in IBD patients compared to controls, which implied an association between histidine and an increased risk of IBD [42]. Therefore, EPA might reduce IBD risk through the regulation of histidine levels. Additionally, since there is competition for shared enzymes and metabolic substrates in the synthesis of omega-3 and omega-6 PUFAs, EPA might also influence the levels of linoleic acid. A previous study indicated that higher levels of linoleic acid, which are involved in the production of proinflammatory mediators, were found in IBD patients compared with controls, thereby implicating an increased risk of IBD [43]. Lower levels of linoleic acid might mediate the protective effects of EPA and IBD. In this study, we showed the causal effects of EPA on α-linolenic acid, linoleic acid, and methylhistidine metabolic pathways and three key metabolites (DHA, linoleic acid, and histidine). These results provide valuable insights into the metabolic mechanism through which EPA influences IBD risk.

Collectively, the increased risk of IBD is primarily associated with higher levels of α-linolenic acid or lower levels of EPA, as the differences in desaturation steps driven by the *FADS2* gene will lead to changes in both upstream α-linolenic acid and downstream EPA concentrations [7]. Thus, the role of the *FADS2* gene is crucial and merits further investigation.

Our study also revealed a massive influence of *FADS2* variants on IBD and CD, but not on UC. Furthermore, we found robust colocalization evidence between omega-3 PUFAs and CD in the *FADS2* gene region, but little colocalization evidence for UC. These findings suggest that the key link between omega-3 PUFAs and IBD is driven by effects in the *FADS2* gene cluster. Several lines of evidence support our observations and indicate that the *FADS2* gene is associated with inflammation [44] and CD risk [45,46]. For instance, the *FADS2* gene regulated immune functions and showed colocalization evidence on PUFAs and CD (posterior probability = 0.94) [45]. In addition, integrated data from metabolomics profiling and experiments revealed the role of *FADS2* against chronic inflammation among CD patients [47]. Therefore, *FADS2* is a crucial gene linking omega-3 PUFAs and IBD risk, particularly in the case of CD. 

Despite the protective role on CD, our study provided little evidence to support the effect of omega-3 PUFAs on UC risk. Previous epidemiological studies also indicated that an increasing dietary intake of EPA or DHA had no association with a decreased risk or maintenance of remission in UC [37,48]. It is possible that inadequate supplementation or absorption resulted in lower concentrations of fatty acids in UC patients, thereby limiting their ability to trigger protective effects. For example, the inflamed colonic mucosa of patients with UC was linked to a significant decrease in EPA [49]. Similarly, a significant reduction in DHA derivatives was observed in active inflammatory UC [50]. As our bidirectional MR analysis showed, genetic liability to UC had an effect on decreased concentrations of DHA. Therefore, whether it is rational for UC patients to increase supplementation of fish oil or enhance intestinal absorption ability is worth further investigation. In contrast, He et al. recently reported that total omega-3 fatty acid had no causal effect on CD, but decreased UC risk using MR [15]. We believe the discrepant association observed for UC in our study compared with theirs was partly driven by the different instrument selection process. After applying a similar instrument selection as our study, He et al. further eliminated SNPs associated with potential confounders between total omega-3 fatty acid and outcomes. This selection process eliminated over half of the genetic variants from the instrument list for total omega-3 fatty acid. He et al. claimed that this selection was used to satisfy the second assumption of MR (exchangeability). However, this assumption suggested that the instruments are not associated with common causes (confounders) of the instrument–outcome association. MR estimates are generally less susceptible to confounders because human DNA is stable across the life course. Therefore, excluding SNPs associated with confounders between total omega-3 and IBD (e.g., body mass index) will reduce the power of the analysis rather than satisfying the exchangeability assumption of MR. In fact, such an overly stringent selection resulted in the deprivation of genetic variants in the *FADS2* region. As mentioned above, the *FADS* gene cluster plays a central role on PUFAs’ metabolism, where genetic effects in the *FADS2* region massively influenced the MR estimates of omega-3 PUFAs on IBD and its subtypes. The effects of total omega-3 fatty acid were found to potentially increase IBD risks after removing the *FADS2* instrument (Figure S2). In summary, the previously reported effect of total omega-3 fatty acid on a lower risk of UC was methodologically arguable and did not align with the evidence from our MR study and other observational studies.

There were several strengths of the present study. First, our study comprehensively explored the causal effects of the different components of omega-3 PUFAs on IBD risk by using a robust MR setting, which reduced bias from residual confounding and excluded reverse causality. Current data contributed to produce informed recommendations based on the relative importance of EPA in preventing IBD. Second, our investigation of the metabolic pathways involving linoleic acid and histidine metabolites provided valuable insights into the mechanisms underlying the effect of EPA on IBD risk, which may have implications for future clinical practice. Third, our findings suggest that supplementation policies should consider the different subtypes of IBD, as EPA demonstrated a significant effect on reducing the risk of CD but not UC, and genetic liability to UC was associated with lower concentrations of DHA. Additionally, we used colocalization methods to thoroughly explore the possibility of a single shared effect signal in the *FADS2* gene region, thus validating the underlying mechanism linking omega-3 PUFAs with CD. 

However, there were some limitations that should be considered when interpreting our findings. First, we used different data sources for the exposure variables. The genetic instruments for total omega-3 were obtained from the UK Biobank study, while instruments for α-linolenic acid, EPA, and DHA were derived from the CHARGE Consortium. Although both datasets involved participants with European ancestry, there could still be potential biases introduced by using different sources. Second, we assumed that the relationships between omega-3 fatty acids and IBD risk were linear. Non-linear relationships were not taken into consideration and further investigation is needed to explore potential non-linear effects. Finally, although we used univariable MR analyses to estimate the effect of each fatty acid, we were unable to directly estimate the effect of EPA-to-DHA ratio. The EPA-to-DHA ratio is considered important in the clinical application of fish oil, and its potential impact on IBD risk merits further exploration. 

## 5. Conclusions

In conclusion, our comprehensive MR analyses identified that EPA was the key component among the omega-3 PUFAs that may exhibit a protective effect on IBD and CD, but not on UC. There was little evidence to support the effect of total omega-3, α-linolenic acid, or DHA on IBD risks. We also provided novel insights into the underlying mechanisms of EPA, which may influence IBD via α-linolenic acid, linoleic acid and methylhistidine metabolic pathways. Furthermore, the FADS2 gene is likely to be a core gene that mediates the effects of omega-3 PUFAs on IBD risk. Based on these findings, our study recommended the supplementation or dietary intake of EPA, rather than α-linolenic acid or DHA, might be beneficial for preventing the onset of IBD. The proposed mediators have provided novel insights into the underlying mechanisms of EPA. More well powered epidemiological studies and clinical trials are needed to explore the potential benefits of high EPA concentration or EPA/DHA in IBD and its subtypes. Moreover, further research is needed to investigate the role of histidine metabolites in the context of IBD.

## Figures and Tables

**Table 1:** Two-sample Mendelian randomization estimations showing the effect of omega-3 PUFAs on inflammatory bowel disease.

Exposure	No. of SNPs	Methods	Estimate			Heterogeneity		Pleiotropy	
			OR	95% CI	*P*	Q	*P_h_*	MR Egger int *P*	MR-PRESSO *P*
Total omega-3 fatty acid	42	IVW	0.94	(0.82, 1.07)	0.35	232.9	<0.001	0.06	<0.001
		MR-Egger	0.83	(0.69, 0.99)	0.05				
		Weighted median	0.85	(0.80, 0.92)	<0.001				
		MR-PRESSO outlier test	0.88	(0.81, 0.95)	0.003				
α-linolenic acid	12	IVW	1.54	(0.72, 3.29)	0.26	46.7	<0.001	0.65	<0.001
		MR-Egger	1.40	(0.58, 3.39)	0.48				
		Weighted median	1.42	(0.89, 2.28)	0.14				
		MR-PRESSO outlier test	1.24	(0.79, 1.95)	0.38				
EPA	23	IVW	0.78	(0.63, 0.98)	0.03	30.8	0.099	0.97	0.099
		MR-Egger	0.78	(0.45, 1.34)	0.37				
		Weighted median	0.59	(0.45, 0.78)	<0.001				
		MR-PRESSO outlier test	NA	NA	NA				
DHA	6	IVW	1.05	(0.86, 1.28)	0.65	21.6	<0.001	0.56	0.012
		MR-Egger	1.20	(0.75, 1.93)	0.49				
		Weighted median	1.12	(0.98, 1.28)	0.09				
		MR-PRESSO outlier test	1.11	(0.99, 1.25)	0.43				

Abbreviations: CI, confidence interval; DHA, docosahexaenoic acid; EPA, eicosapentaenoic acid; Egger int, egger intercept; IVW, inverse variance weighted; MR, Mendelian randomization; OR, odds ratio; PUFAs, polyunsaturated fatty acids; *P_h_*, *p*-value for heterogeneity.

**Table 2:** Bidirectional Mendelian randomization estimates for causal effects of genetic liability to IBD, CD, and UC on the levels of omega-3 PUFAs.

Exposure	No. of SNPs	Outcome	No. of SNPs	IVW			Heterogeneity		Pleiotropy	
				Beta	95% CI	*P*	Q	*P_h_*	MR Egger int *P*	MR-PRESSO*P*
IBD	117	Total omega-3 fatty acid	105	−0.002	(−0.012, 0.009)	0.76	200.7	<0.001	0.86	<0.001
		α-linolenic acid	39	−0.001	(−0.004, 0.001)	0.36	35.9	0.57	0.94	0.416
		EPA	39	0.001	(−0.019, 0.020)	0.92	57.9	0.02	0.87	0.009
		DHA	39	−0.010	(−0.059, 0.040)	0.71	47.7	0.13	0.69	0.036
CD	89	Total omega-3 fatty acid	83	0.004	(−0.005, 0.013)	0.39	174.5	<0.001	0.28	<0.001
		α-linolenic acid	28	−0.001	(−0.003, 0.001)	0.28	28.7	0.38	0.41	0.463
		EPA	28	0.011	(−0.005, 0.026)	0.17	46.4	0.01	0.85	0.012
		DHA	28	0.029	(−0.011, 0.070)	0.15	39.3	0.06	0.81	0.090
UC	62	Total omega-3 fatty acid	53	−0.005	(−0.018, 0.008)	0.45	131.0	<0.001	0.27	<0.001
		α-linolenic acid	27	0.002	(−0.001, 0.004)	0.23	28.5	0.34	0.34	0.446
		EPA	27	−0.005	(−0.021, 0.010)	0.50	26.5	0.44	0.38	0.088
		DHA	27	−0.045	(−0.089, −0.002)	0.04	26.7	0.42	0.61	0.095

Abbreviations: CI, confidence interval; CD, Crohn’s disease; DHA, docosahexaenoic acid; EPA, eicosapentaenoic acid; Egger int, Egger intercept; IBD, inflammatory bowel disease; IVW, inverse variance weighted; MR, Mendelian randomization; PUFAs, polyunsaturated fatty acids; *P_h_*, *p*-value for heterogeneity; UC, ulcerative colitis.

## Data Availability

The summary statistics of total omega-3 fatty acid were obtained from UK Biobank study at https://doi.org/10.1186/s12916-022-02399-w, accessed on 6 November 2022, and instruments for α-linolenic acid, eicosapentaenoic acid, and docosahexaenoic acid were derived from Cohorts for Heart and Aging Research in Genomic Epidemiology Consortium at https://www.chargeconsortium.com/main/results, accessed on 6 November 2022. Genetic association estimates for inflammatory bowel disease were obtained from the study by the International Inflammatory Bowel Disease Genetics Consortium (IIBDGC) at https://doi.org/10.1038/ng.3760, accessed on 6 November 2022. The full summary statistics of the circulating metabolites were derived from the IEU OpenGWAS database at https://gwas.mrcieu.ac.uk/, accessed on 6 November 2022.

## Supplementary Materials

The following supporting information can be downloaded at: https://www.mdpi.com/article/10.3390/metabo13101041/s1, Figure S1: Selection process for the data included in the study; Figure S2: Leave one out plots of the causal effects of omega-3 polyunsaturated fatty acids on inflammatory bowel disease, Crohn’s disease, and ulcerative colitis showing inverse variance weighted estimates after omitting each SNP; Table S1: Data sources of genome-wide association studies included in the Mendelian randomization analysis; Table S2: Statistics used to assess instrument strength; Table S3: Sensitivity analyses used to assess causal effects of omega-3 polyunsaturated fatty acids on the risk of Crohn’s disease, and ulcerative colitis.
