# Supplementary material for: Evaluating the Effects of Omega-3 Polyunsaturated Fatty Acids on Inflammatory Bowel Disease via Circulating Metabolites: A Mediation Mendelian Randomization Study
Source: Metabolites. 2023 Sep 28;13(10):1041. doi: 10.3390/metabo13101041 (PMC10608743; doi:10.3390/metabo13101041)
Supplement: Supplementary file 1 [file metabolites-13-01041-s001.zip › metabolites-2524759-supplementary.pdf]

Online Supplementary Materials

Figure S1. Selection process for the data included in the study.

Figure S2. Leave one out plots of the causal effects of omega-3 polyunsaturated fatty acids on inflammatory bowel disease, Crohn's disease, and ulcerative colitis showing inverse variance weighted estimates after omitting each SNP.

Table S1. Data sources of genome-wide association studies included in the Mendelian randomization analysis.

Table S2. Statistics used to assess instrument strength.

Table S3. Sensitivity analyses used to assess causal effects of omega-3 polyunsaturated fatty acids on the risk of Crohn's disease, and ulcerative colitis.

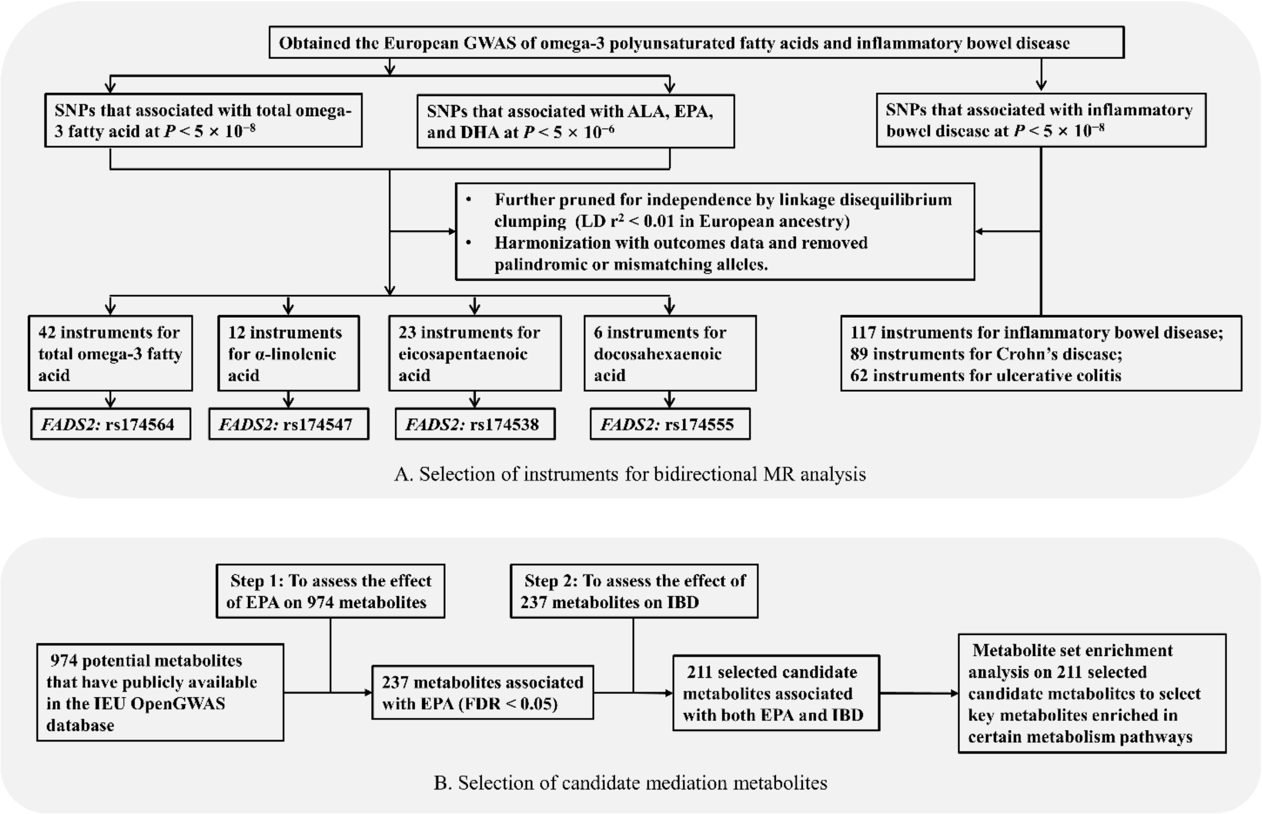

Supplementary Figure 1

Figure S1. Selection process for the data included in the study. ALA, α-linolenic acid; DHA, docosahexaenoic acid; EPA, eicosapentaenoic acid; *FADS2, fatty acid desaturase 2*; GWAS, genome-wide association study; MR, Mendelian randomization; SNPs, Single-nucleotide polymorphisms.

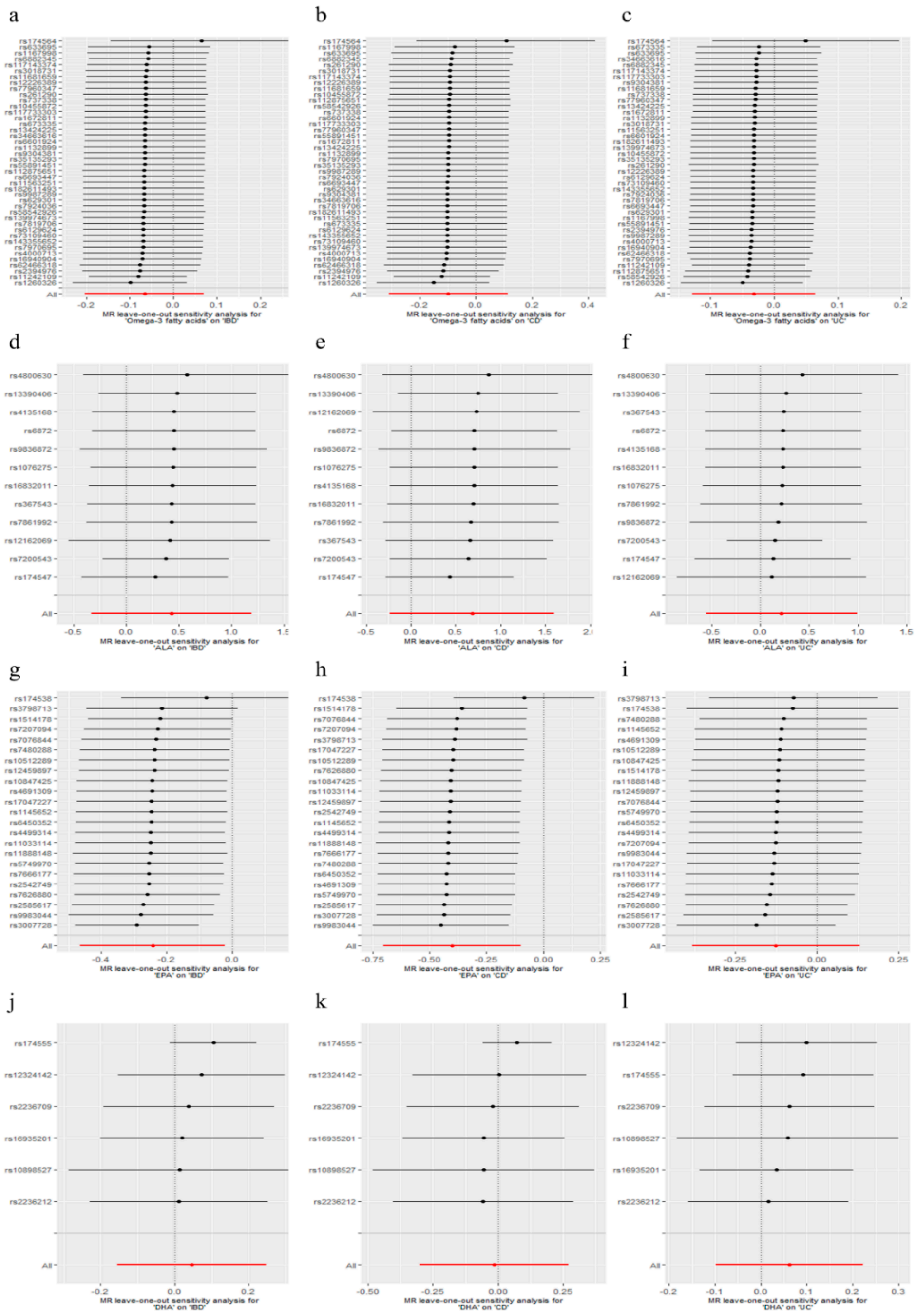

Supplementary Figure S2

Figure S2. Leave one out plots of the causal effects of omega-3 polyunsaturated fatty acids on inflammatory bowel disease, Crohn's disease, and ulcerative colitis showing inverse variance weighted estimates after omitting each SNP. Each black point represents the IVW MR method applied to estimate the causal effect of omega-3 PUFAs on IBD excluding that particular variant from the analysis. The red point depicts the IVW estimate using all SNPs.

Table S1. Data sources of genome-wide association studies included in the Mendelian randomization analysis.

Instrument	Phenotype	Reference	Consortium	Sample size	Ancestry
Instrument-exposure information	Total omega-3 fatty acid	Borges MC et al [17]	UK Biobank	up to 114,999	European
	α-linolenic acid	Lemaitre et al [18]	CHARGE Consortium	up to 8866	European
	Eicosapentaenoic acid				
	Docosahexaenoic acid				
Instrument-outcome information	Inflammatory bowel disease	de Lange et al [19]	International Inflammatory Bowel Disease Genetics Consortium	Cases=25,042 controls=34,915	European
	Crohn's disease			Cases=12,194 controls=28,072	European
	Ulcerative colitis			Cases=12,366 controls=33,609	European

Table S2. Statistics used to assess instrument strength.

Exposure	Outcome	No. of SNPs	*F*	*F* (*F* – 1) / *F*	I^2GX
Total omega-3 fatty acid	IBD/CD/UC	42	262.21	0.996	0.99
ALA		12	47.74	0.950	0.94
EPA		23	34.56	0.972	0.84
DHA		6	29.82	0.956	0.82

Abbreviations: ALA, α-linolenic acid; CD, Crohn's disease; DHA, docosahexaenoic acid; EPA, eicosapentaenoic acid; IBD, inflammatory bowel disease; UC, ulcerative colitis.

Table S3. Sensitivity analyses used to assess causal effects of omega-3 polyunsaturated fatty acids on the risk of Crohn's disease, and ulcerative colitis.

Exposure	Outcome	MR-Egger		Weighted median		Heterogeneity		Pleiotropy	
		OR (95% CI)	*p*	OR (95% CI)	*p*	Q	P_h	MR Egger int *p* value	MR-PRESSO *p* value
Total omega-3 fatty acid	CD	0.74 (0.56, 0.98)	0.04	0.79 (0.72, 0.86)	<0.001	331.1	<0.001	0.04	<0.001
ALA		1.70 (0.59, 4.92)	0.35	1.72 (0.93, 3.17)	0.08	41.1	<0.001	0.56	0.002
EPA		0.52 (0.25, 1.06)	0.09	0.59 (0.39, 0.88)	0.01	33.9	0.05	0.45	0.03
DHA		1.29 (0.68, 2.47)	0.48	1.05 (0.89, 1.24)	0.56	26.1	<0.001	0.41	0.005
Total omega-3 fatty acid	UC	0.91 (0.80, 1.04)	0.17	0.93 (0.85, 1.02)	0.12	71.4	0.002	0.19	<0.001
ALA		1.15 (0.46, 2.86)	0.77	1.35 (0.73, 2.49)	0.33	30.2	0.002	0.73	0.014
EPA		1.05 (0.56, 1.94)	0.89	0.81 (0.56, 1.17)	0.22	24.9	0.30	0.56	0.35
DHA		1.08 (0.73, 1.60)	0.73	1.08 (0.91, 1.28)	0.39	8.5	0.13	0.94	0.23

Abbreviations: ALA, α-linolenic acid; CI, confidence interval; CD, Crohn’s disease; DHA, docosahexaenoic acid; EPA, eicosapentaenoic acid; Egger int, egger intercept; MR, Mendelian randomization; OR, odds ratio; P_h, *P*-value for heterogeneity; UC, ulcerative colitis.
